# Gaps of Medication Treatment Management Between Guidelines and Real-World for Inpatients With Type 2 Diabetes in China From Pharmacist’s Perspective

**DOI:** 10.3389/fendo.2022.900114

**Published:** 2022-05-30

**Authors:** Zhi-Hui Song, Xing-Long Wang, Xiao-Feng Wang, Jing Liu, Sang-Quzhen Luo, Shan-Shan Xu, Xiao Cheng, Jie Bai, Li-ming Dong, Chao Zhang, Jian-Bo Zhou

**Affiliations:** ^1^ Department of Pharmacy, Beijing Tongren Hospital, Capital Medical University, Beijing, China; ^2^ Department of Clinical Pharmacy, Xilingol Mongolian Hospital, Xilinhot, China; ^3^ Department of Pharmacy, Traditional Chinese Medicine Hospital Affiliated to Xinjiang Medical University, Urumqi, China; ^4^ Department of Pharmacy, Lhasa People’s Hospital, Lhasa, China; ^5^ Department of Endocrinology, Beijing Tongren Hospital, Capital Medical University, Beijing, China

**Keywords:** diabetes, real-word, medication treatment management, guidelines, pharmacist

## Abstract

**Background:**

The prevalence of diabetes mellitus remains high in China, and more cardiovascular and cerebrovascular adverse events due to diabetes mellitus are likely to occur in the future.

**Objective:**

To analyze the gap between the current pharmacotherapy management and the guidelines for inpatients with type 2 diabetes mellitus from the perspective of pharmacists so as to provide a reference for optimal pharmacotherapy management methods and models for patients with type 2 diabetes mellitus.

**Methods:**

The study was a cross-sectional observational study. The study was conducted by investigating and analyzing the use of glucose-lowering drugs, adjustment of blood pressure management strategy, lipid management, weight management, and application of antiplatelet drugs in type 2 diabetes inpatients.

**Results:**

A total of 1086 patients with type 2 diabetes were included. Metformin, glycosidase inhibitors, and basal insulin were the most used among type 2 diabetes inpatients. The use of SGLT-2, GLP-1 RAs, DPP-4, and metformin all showed significant increase. SGLT-2 inhibitors (SGLT-2i) showed the fastest increase from 2020 to 2021 (14.5% vs. 39.6%); However, the application rate of SGLT-2i was low among patients with combined ASCVD, renal insufficiency, and diabetic nephropathy (46.4%, 40.9%, and 45.8% respectively). For patients with substandard blood pressure at admission, the average rate of intervention by endocrinologists for adjusting the antihypertensive regimen during hospitalization was 55.6%, and the application rate of ACEI/ARB drugs reached 64.4%. The application rate of statins among patients with type 2 diabetes was still relatively high, at 78.8%. However, the overall intervention rate for patients with suboptimal LDL-c was only 24.1%. The application rate of antiplatelet agents for patients with ASCVD was 77.6%, which was higher than that for patients without ASCVD.

**Conclusion:**

There is still a gap between the practice of medication treatment management of Chinese inpatients with type 2 diabetes and the guidelines, especially in the application of GLP-1RAs and SGLT-2i in patients with concomitant ASCVD, diabetic nephropathy, and renal insufficiency. Meanwhile, physicians and pharmacists should pay more attention on achieving blood pressure and LDL-c standards in type 2 diabetic patients and provide timely interventions.

## Introduction

According to the latest data from the International Diabetes Federation (IDF), about 463 million adults (20–79 years old) worldwide had diabetes in 2019 (1 in 11 people) and is expected to reach 700.2 million by 2045. The number of people with diabetes in China is now the highest in the world, with an incidence of 116.4 million people and a prevalence of 12.8% (1). Type 2 diabetes (T2DM) accounts for more than 90% of the diabetic population while type 1 diabetes (T1DM) and other types of diabetes are rare (1, 2). Atherosclerotic cardiovascular disease (ASCVD)-defined as coronary heart disease (CHD), cerebrovascular disease, or peripheral arterial disease presumed to be of atherosclerotic origin-is the leading cause of morbidity and mortality in individuals with diabetes (2–4). Glycemic control alone has a limited role in reducing the risk of ASCVD and the integrated management of multiple risk factors such as glycemia, blood pressure, lipids, and obesity, as well as the combination of appropriate antiplatelet therapy is crucial to minimize the risk of cardiovascular events and mortality (3, 5, 6).

At present, there are a variety of antidiabetic drugs in addition to the hypoglycemic effect, which also have many cardiac and renal benefits, such as SGLT-2 inhibitors (SGLT-2i) (7–14) and GLP-1 receptor agonists (GLP-1 RAs) (14, 15), which have been widely recommended for patients with ASCVD or chronic kidney disease (CKD) (3, 4, 16, 17). Metformin (18–20) has been shown to help delay the progression of diabetic macrovascular complications, in a small number of studies but the studies on DPP-4 inhibitors and other antidiabetic drugs on cardiovascular benefits are mostly neutral (21).

Currently, the involvement of pharmacists in the management of pharmacotherapy in diabetic patients is beneficial in improving patient medication adherence and glycemic compliance, and reducing rehospitalization rates (22–25). Endocrine clinical pharmacists have also worked in many hospital endocrine departments to assist physicians in the management of medication therapy for patients with diabetes. However, the achievement of the relevant indicators in diabetic patients is still unsatisfactory in terms of the achievement of the indicators in diabetic patients.

Therefore, a descriptive analysis of pharmacotherapy management in hospitalized patients with T2DM involving clinical pharmacists is presented to assess the gap between medication treatment management (MTM) of T2DM and guidelines in the real world.

## Methods

### Patients

Patients with T2DM who received treatment at the Department of Endocrinology, Beijing Tongren Hospital, Capital Medical University, from January 2020 to December 2021. The inclusion criteria were a clear diagnosis of T2DM using diagnostic criteria from the Chinese Guidelines for the Prevention and Treatment of Type 2 Diabetes Mellitus (2020 version) (2), a time detection of plasma glucose level ≥ 11.1 mmol·L^-1^, fasting blood glucose (FBG) ≥ 7.0 mmol-L^-1^ or oral glucose tolerance test (OGTT) 2h glucose ≥ 11.1 mmol-L ^-1^ ≥18 years old or above, complete clinical data of patients present in the hospital information system and electronic medical record system. Patients were excluded if they were pregnant or lactating.

We collected data on age, gender, body mass index (BMI), co-morbidities, admission treatment, relevant examination and laboratory results. The patients were divided into two groups, those with ASCVD and those without ASCVD, and the medications and related indexes were statistically analyzed in both groups. ASCVD was defined as a history of coronary heart disease, cerebral infarction, revascularization, or peripheral arterial disease.

Blood glucose, blood pressure, and lipid targets were set according to the Chinese Guidelines for the Prevention and Treatment of Type 2 Diabetes Mellitus (2020 Edition) (2). The blood glucose standard was defined as glycosylated hemoglobin (HbA1c) <7%, the blood pressure standard was defined as blood pressure <130/80 mmHg and the low-density lipoprotein cholesterol (LCL-C) standard was defined as LCL-C level <1.8 mmol-L^-1^ in patients with a history of coronary heart disease and cerebral infarction, and LDL-C level <2.6 mmol-L^-1^ in patients without a history of coronary heart disease and cerebral infarction.

### Statistics

The data were analyzed using SPSS22.0 statistical software; the measurement data were expressed as (x ± s), where “x” means the average and “s” represents standard deviation. Independent Sample Student’s t-test was used for comparison between groups. The homogeneity of variance test was used to test homogeneity of data and when P > 0.05 there is homogeneity of variance but when P ≦0.05, the variance is not homogeneous. The count data were expressed as cases (%), and the χ^2^-test was used for comparison between groups. A P value < 0.05 indicated that the differences were statistically significant.

## Results

A total of 1090 patients with T2DM were admitted to the Beijing Tongren Hospital according to the electronic medical record system between 2020 and 2021. Four patients were excluded because two of the patients were not diagnosed with T2DM and two were discharged after only a day of hospitalization with incomplete data information. Therefore, 1086 patients with T2DM were finally included. Details of sex ratio, BMI, smoking status, diabetes course, concomitant disease, blood glucose, blood pressure, lipids, and comorbidities at the time of admission are shown in **Table 1**.

**Table 1:** Baseline characteristics of inpatients with type 2 diabetes.

Characteristic	2020	2021	Total
Cases/cases (%)	420	666	1086
Age- yr	59 ± 12	55 ± 13	57 ± 12
Female sex — no. (%)	255 (60.7)	272 (55.9)	627(57.7)
Current smoker			
smoker— no. (%)	167 (39.8)	221 (33.2)	388 (35.7)
non-smoker— no. (%)	253 (60.2)	445 (66.8)	698 (64.3)
Duration of diabetes— yr.	13.8 ± 8.8	12.4 ± 8.8	13.1 ± 8.8
Body-mass index (kg/m^2^).	25.8 ± 3.5	25.9 ± 3.6	25.8 ± 3.6
With ASCVD			
History of coronary heart disease— no. (%)	95 (22.6)	169 (25.4)	264 (24.3)
History of cerebral infarction— no. (%)	81 (19.3)	78 (11.7)	159 (14.6)
Revascularization— no. (%)	53 (12.6)	64 (9.6)	117 (10.8)
Peripheral artery disease— no. (%)	13 (3.1)	16 (2.4)	29 (2.7)
Complications			
Hypertension— no. (%)	276 (65.7)	403 (60.5)	679 (62.5)
Dyslipidemia — no. (%)	328 (78.1)	469 (70.4)	797 (73.4)
Hyperuricemia— no. (%)	94 (22.4)	126 (18.9)	220 (20.3)
Thyroid nodule(thyroid cancer )— no. (%)	254 (60.5)	268 (40.2)	522 (48.1)
Renal insufficiency— no. (%)	118 (28.1)	239 (35.9)	357 (32.9)
Diabetic Nephropathy — no. (%)	118 (28.1)	466 (70)	584 (53.8)
Diabetic retinopathy— no. (%)	142 (33.8)	409 (61.4)	551 (50.7)
Blood glucose test on admission			
FBG (fasting blood glucose)	8.2 ± 2.9	7.93 ± 2.8	8.05 ± 2.8
HbA1c (%)	8.8 ± 2.4	8.9 ± 2.0	8.85 ± 2.2
Blood lipid testing on admission			
TC/(mmol·L^-1^)	4.4 ± 1.2	4.5 ± 1.1	4.45 ± 1.1
TG/(mmol·L^-1^)	1.8 ± 1.1	1.9 ± 1.3	1.85 ± 1.2
HDL-C/(mmol·L^-1^)	1.1 ± 0.4	1.1 ± 0.5	1.1 ± 0.4
LDL-C/(mmol·L^-1^)	2.7 ± 2.7	2.6 ± 0.9	2.65 ± 1.8
Blood pressure test on admission			
Systolic pressure (mmHg)	130 ± 16	128 ± 17	129 ± 16
Diastolic blood pressure (mmHg)	78 ± 14	76 ± 10	77 ± 7

### Blood Glucose Management and Medication Use

**Figure 1:**
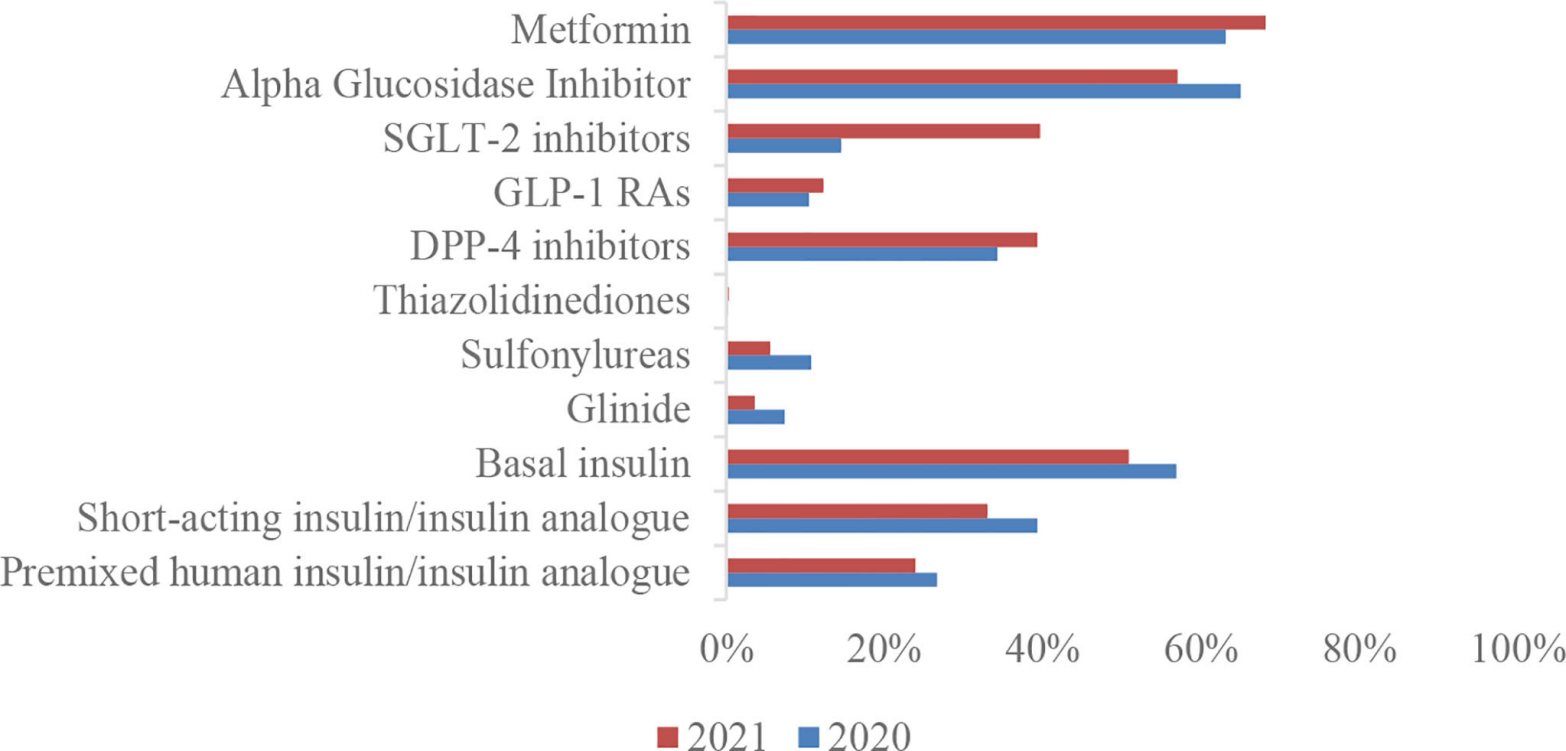
Application rates of different categories of hypoglycemic agents of inpatients with type 2 diabetes.

The inpatient glucose-lowering drug regimen was dominated by insulin intensive therapy combined with other glucose-lowering drug regimens followed by dual drug combinations and metformin, glycosidase inhibitors, and basal insulin were the most used among T2DM inpatients (**Table 2**). Compared to 2020, the application of SGLT-2is, GLP-1 RAs DPP-4 inhibitors, and metformin all increased, while several other categories of hypoglycemic agents were decreasing. SGLT-2i showed the fastest increase in application rate from 2020 to 2021 (39.6% vs. 14.5%) (**Table 3**, **Figure 1**).

**Table 2:** Diabetes medication regimen of inpatients with type 2 diabetes.

Diabetes medication regimen	2020	2021	Total
Cases/cases (%)	420	666	1086
Monotherapy	91 (21.7%)	412 (61.9%)	503 (46.3%)
Metformin	41(9.8%)	119 (17.9%)	160 (14.7%)
DPP-4 inhibitors	10 (2.4%)	54 (8.1%)	64 (5.9%)
GLP-1 RA	3 (0.7%)	22 (3.3%)	25 (2.3%)
SGLT - 2i	5 (1.2%)	69 (10.4%)	74 (6.8%)
Glycosidase inhibitors	23 (5.5%)	74 (11.1%)	97 (8.9%)
Sulphonylureas	4 (1%)	15 (2.3%)	19 (1.7%)
Glycinide	4 (1%)	8 (1.2%)	12 (1.1%)
Insulin	1 (0.2%)	51 (7.7%)	52 (4.8%)
Double drugs treatment	130 (31%)	223 (33.5%)	353 (32.5%)
Metformin +others	76 (18.1%)	123 (18.5%)	199 (18.3%)
(GLP-1 RAs or SGLT-2i)+others	27 (6.4%)	80 (12%)	107 (9.9%)
Other double medicine treatment	34 (8.1%)	44 (6.6%)	78 (7.2%)
Three drugs treatment	15 (3.6%)	101 (15.2%)	116 (10.7%)
Metformin +others	12 (2.9%)	50 (7.5%)	62 (5.7%)
(GLP-1 RAs or SGLT-2i)+others	1 (0.2%)	45 (6.8%)	46 (4.2%)
Other three medicine treatment	2 (0.5%)	37 (5.6%)	39 (3.6%)
Insulin intensive therapy +others	230 (54.8%)	334 (50.2%)	564 (51.9%)
Insulin intensive therapy+ metformin +others	30 (7.1%)	98 (14.7%)	128 (11.8%)
Insulin intensive therapy+(GLP-1 RAs or SGLT-2i) +others	34 (8.1%)	164 (24.6%)	198 (18.2%)
Others (In addition to insulin, a variety of hypoglycemic drugs combined)	15 (3.6%)	43 (6.5%)	58 (5.3%)
Metformin included	12 (2.9%)	37 (5.6%)	49 (4.5%)
GLP-1 RAs or SGLT-2i included	10 (2.4%)	35 (5.3%)	45 (4.1%)

**Table 3:** Application rates of different categories of hypoglycemic agents of inpatients with type 2 diabetes.

Hypoglycemic agent categories	2020	2021	Total
Metformin	63.1%	68.2%	66.2%
Alpha Glucosidase Inhibitor	65.0%	57.1%	60.1%
SGLT-2 inhibitors	14.5%	39.6%	29.9%
GLP-1 RAs	10.5%	12.3%	11.6%
DPP-4 inhibitors	34.3%	39.3%	37.4%
Thiazolidinediones	0.2%	0.3%	0.3%
Sulfonylureas	10.7%	5.6%	7.6%
Glinide	7.4%	3.6%	5.1%
Basal insulin	56.9%	50.9%	53.2%
Short-acting insulin/insulin analogue	39.3%	33.0%	35.5%
Premixed human insulin/insulin analogue	26.7%	23.9%	25.0%

**Figure 2:**
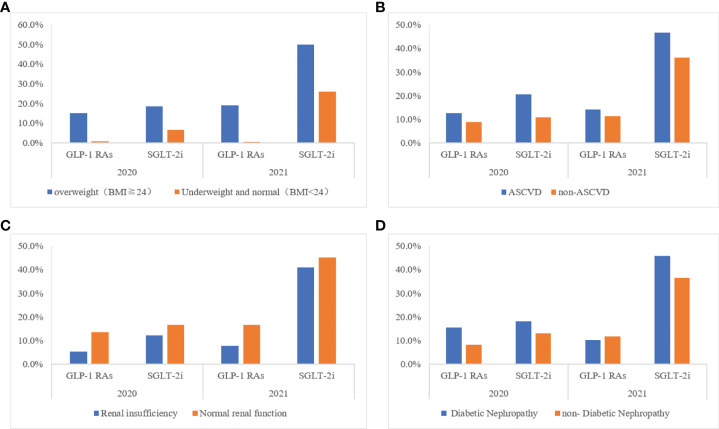
Application rates of GLP-1 RAs/SGLT-2i of inpatients with type 2 diabetes in different clinical situations **(A)** Different body size population; **(B)** With or no cardiovascular disease; **(C)** With or no renal insufficiency; **(D)** With or no Diabetic Nephropathy.

In addition, GLP-1RAs and SGLT-2i application rates are higher in patients that are overweight (24 kg/m^2^≦BMI<28 kg/m^2^) or obese (BMI≧28 kg/m^2^), inpatients with ASCVD, and patients with diabetic nephropathy; however, the application rates in patients with renal insufficiency (Ccr 45–90 ml/min/1.73m^2^) were lower than those with normal renal function (Ccr >90 ml/min/1.73m^2^) (**Figures 2A–D**). Moreover, the application rate of SGLT-2, is only 46.4%, 40.9%, and 45.8% among patients with ASCVD, renal insufficiency (Ccr 45–90ml/min), and diabetic nephropathy respectively in 2021, and the application rate of GLP-1 is even less (**Table 4**).

**Table 4:** Application rates of GLP-1 RAs/SGLT-2i of inpatients with type 2 diabetes in different clinical situations.

The clinical situation		2020	2021		2020	2021
	Total (n)	GLP-1RAs	GLP-1RAs	Total	SGLT-2i	SGLT-2i
Body-mass index						
BMI≧24kg/m^2^— no. (%)	276	42 (15.2)	64 (19)	336	51 (18.5)	168 (50)
BMI<24kg/m^2^— no. (%)	138	1 (0.7)	1 (0.6)	162	9 (6.5)	42 (25.9)
* P*		0.89			0.20	
History of ASCVD						
ASCVD— no. (%)	151	19 (12.6)	31 (14.2)	219	31 (20.5)	102 (46.6)
non-ASCVD— no. (%)	269	24 (8.9)	51 (11.4)	446	29 (10.8)	161 (36.1)
* P*		0.47			0.21	
Renal function						
renal insufficiency — no. (%)	147	8 (5.4)	15 (7.8)	193	18 (12.2)	79 (40.9)
normal renal function.— no. (%)	258	35 (13.6)	59 (16.6)	356	43 (16.7)	161 (45.2)
* P*		0.70			0.012	
Diabetic Nephropathy						
with Diabetic Nephropathy— no. (%)	116	18 (15.5)	6 (10.2)	59	21 (18.1)	27 (45.8)
non- Diabetic Nephropathy— no. (%)	306	25 (8.2)	62 (11.7)	528	40 (13.1)	193 (36.6)
* P*		0.83			0.039	

### Blood Pressure Management and Medication Use

Patients with T2DM have a significantly higher rate of blood pressure compliance at discharge compared to that at admission (**Table 5**). For patients with substandard blood pressure on admission, the percentage of interventions by endocrinologists during hospitalization to adjust the antihypertensive regimen was 55.6% on average, but the intervention rate decreased in 2021. In addition, ACEI/ARB is the most commonly used, with an application rate of 64.4%.

**Table 5:** Blood pressure and medication of type 2 diabetes inpatients.

	2020		2021		Total
	admission	discharge	admission	discharge	
Systolic pressure (mmHg)	130 ± 16	124 ± 14	128 ± 17	123 ± 11	
Diastolic blood pressure (mmHg)	78 ± 14	75 ± 12	76 ± 10	75 ± 14	
Heart rate(times/min)	76 ± 11	72 ± 8	77 ± 10	74 ± 8	
Blood pressure reaching standard— no. (%)	34.8% (146/420)	57.1% (240/420)	43.0% (286/666)	60.3% (401/666)	59.1%
Intervention rate for patients not up to blood pressure standard—% (no.)	73.7% (202/274)		43.0% (163/380)		55.6%
ACEI/ARB Usage rate (%)—%(no.)	67.4% (215/319)		62.2% (275/442)		64.4%

### Lipid Management and Medication Use

**Figure 3:**
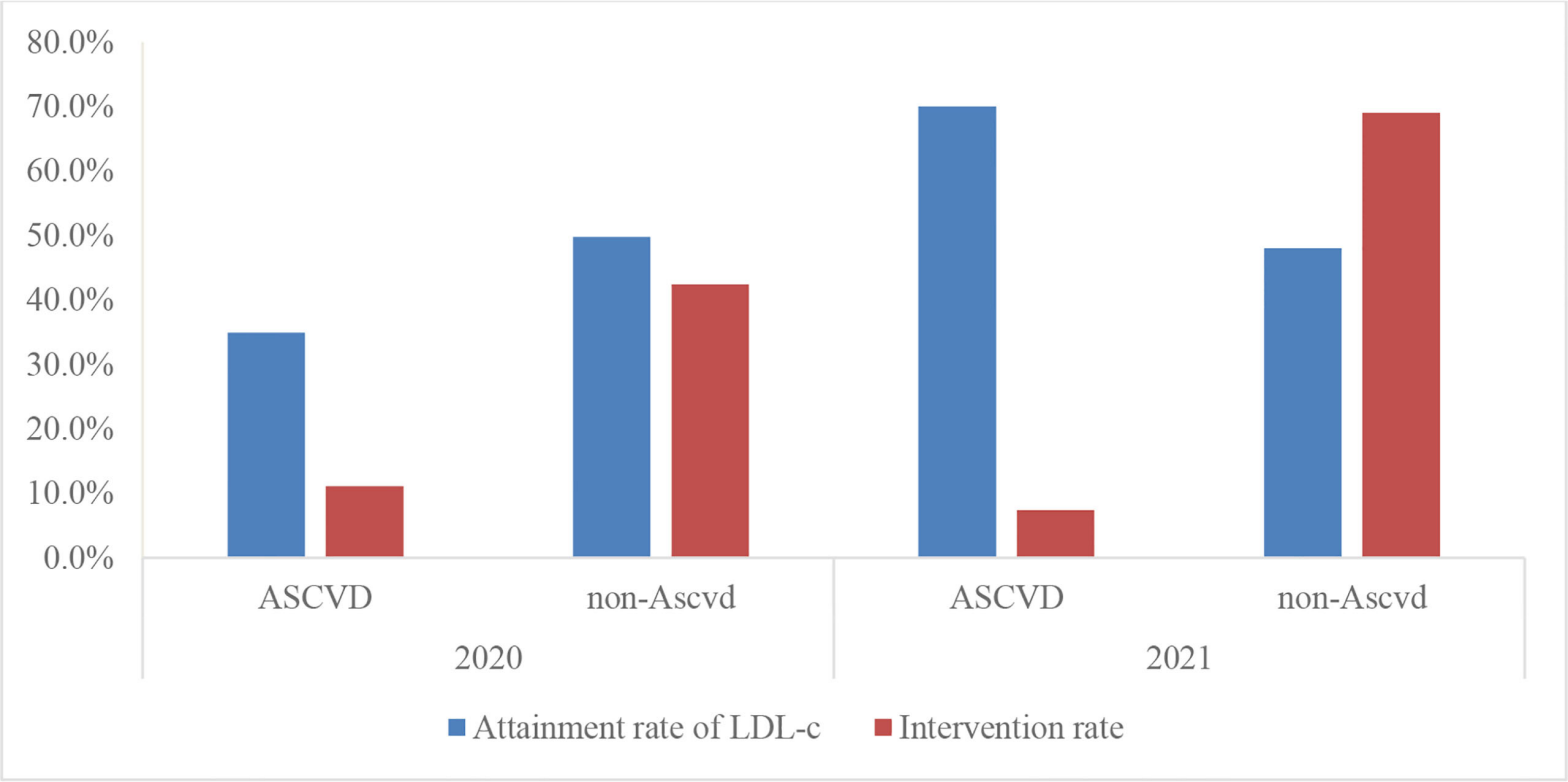
Attainment rate of LDL-c and intervention rate of type 2 diabetes in patients with or without ASCVD.

There were no lipid test results in 3 of 1086 patients with T2DM, so some data included only 1083 patients. These patients had a compliance rate of 75.3%, 58.3%, 38.8%, and 43.3% for total cholesterol, triglycerides, HDL cholesterol, and LDL cholesterol, respectively. Thus, the attainment rate of LDL cholesterol, which is the most important in lipid management, was low (**Table 6**, **Figure 3**). Moreover, the LDL cholesterol compliance rate was lower in patients with ASCVD than in those without ASCVD (33.2% vs. 48.7%) (**Figure 3**). In addition, the overall intervention rate was only 24.1% for patients with non-compliant LDL cholesterol, and similarly, the LDL cholesterol intervention rate was still lower in patients with ASCVD than in patients without ASCVD (8.9% vs. 34.4%). On a better note, however, the statin use rate was still relatively high at 78.8% among all patients using lipid-lowering drugs. However, it is clear that the rate of LDL cholesterol compliance in patients with T2DM in 2021, as well as the rate of intervention for patients not meeting the standard, has decreased.

**Table 6:** Blood lipid and medication of type 2 diabetes inpatients.

	2020	2021	Total
Blood lipid profile at admission	419	664	1083
Attainment rate of TC —% (n)	75.9 (318)	75.0 (498)	75.3 (816)
Attainment rate of TG —% (n)	60.4 (253)	56.9 (378)	58.3 (631)
Attainment rate of HDL-c —% (n)	37.5 (157)	39.6 (263)	38.8 (420)
Attainment rate of LDL-c —% (n)	44.4 (186)	42.6 (283)	43.3 (469)
ASCVD —% (n)	34.9 (53)	32.1 (70)	33.2 (123)
non-ASCVD —% (n)	49.8 (133)	48.0 (214)	48.7 (347)
Intervention rate for patients not up to LDL-c standard —% (n)	11.1 (11)	7.4 (11)	8.9 (22)
ASCVD —% (n)	42.4 (57)	29.7 (69)	34.4 (126)
non-ASCVD —% (n)	29.2 (68)	21.0 (80)	24.1 (148)
The rate of statin use—% (n)	78.3 (303)	79.1 (444)	78.8 (747)

### Antiplatelet Drugs

Antiplatelet agents were prescribed in 503 of 1086 patients (46.3%). Among them, the application rate of antiplatelet drugs among patients with ASCVD (77.6%) was higher than the application rate among patients without ASCVD (30.2%), but the overall antiplatelet drug application rate decreased in 2021, compared with 2020. In terms of antiplatelet drug selection, single-agent aspirin remains the most commonly used antiplatelet therapy drug (**Table 7**).

**Table 7:** Application of antiplatelet drugs of type 2 diabetic inpatients with or without ASCVD.

	2020	2021	Total
ASCVD (n)	152	218	370
Application of antiplatelet drugs—% (n)	83.6 (127)	73.1 (160)	77.6 (287)
aspirin—% (n)	65.1 (99)	50.7 (111)	56.8 (210)
clopidogrel/ticagrelor—% (n)	11.8 (18)	11 (24)	11.4 (42)
aspirin+ (clopidogrel/ticagrelor)—% (n)	14.5 (22)	11 (24)	12.4 (46)
others—% (n)	0	0	0
non-ASCVD (no)	268	448	716
Application of antiplatelet drugs—% (n)	40.3 (108)	24.1 (108)	30.2 (216)
aspirin—% (n)	33.2 (89)	22.3 (100)	26.4 (189)
clopidogrel/ticagrelor—% (n)	4.9 (13)	1.3 (6)	2.6 (19)
aspirin+ (clopidogrel/ticagrelor)—% (n)	1.9 (5)	0.4 (2)	1.0 (7)
others—% (n)	0	0	0

## Discussion

From this study, it is evident that there is a large gap between the practice of medication therapeutic management and the guidelines for Chinese inpatients with T2DM. This is reflected by the application rate of GLP-1 RAs and SGLT-2i increasing but is still generally low, the proportion of interventions by endocrinologists for patients with substandard blood pressure to adjust the antihypertensive regimen during hospitalization is low, suggesting a deficiency in clinical patient blood pressure management, the overall intervention rate for patients with substandard LDL cholesterol was too low, suggesting insufficient clinical attention to LDL cholesterol standard attainment, especially for patients with ASCVD and inadequate use of antiplatelet agents in patients with ASCVD. Evidence-based medicine guidelines are increasingly published and sanctioned but may have low adherence (26, 27). Several previous studies investigating the attainment of cardiovascular risk factors in outpatients with diabetes have shown low rates of attainment of glucose, blood pressure, and lipids in outpatients (28, 29), the triple control rate for glycemia, blood pressure, and lipids was 11.2% in China (28) and 24% in the US (29). A meta-analysis, which assessed the achievements of targets according to different guidelines in different countries, reported that the achievements rates were 42.8% for glycemic control, 29% for blood pressure, 49.2% for LDL cholesterol (30). For inpatients, the percentages of patients who met the Chinese Diabetes Society goals—HbA1c <7%, blood pressure <130/80 mmHg, normal lipids, and all three goals—were 30.5, 16.2, 8.0, and 0.9% in 2017 (31), respectively. But there are few studies about the gaps in treatment medication between real-world and guidelines.

Why is there such a large gap? Although, SGLT-2i with GLP-1 RAs in diabetic patients, especially those with ASCVD, are recommended by several guidelines (2–6), they are expensive and many patients, especially those without medical insurance, are restricted. Moreover, since China is currently implementing a Diagnosis Related Groups (DRG) payment policy, the conditions for medical insurance reimbursement for the use of GLP-1RAs or SGLT-2i are relatively strict, which also greatly limits the use in patients with medical insurance. On the other hand, for safety reasons, many physicians conservatively prescribe these drugs are they are too concerned about adverse reactions. For example, some doctors will stop treatment with SGLT-2i if a patient has a positive urinary ketone body, severe lower extremity atherosclerosis or previous urinary tract infections. This is due to the risk of ketoacidosis, increased risk of amputation or increased recurrent urinary tract infection.

The main reason for the gap in the guidelines in blood pressure management, lipid management, and antiplatelet drug use may be due to the lack of awareness of integrated management among endocrinologists, or the fact that although they are aware of integrated management, they do not pay much attention to the achieving blood pressure and lipid standards. Because clinical subspecialties are very detailed, blood pressure management, lipid management, and antiplatelet drug use are all grouped into cardiovascular or neurology departments, and endocrinology departments pay more attention to blood glucose management, blood pressure, and lipids while neglecting the control of other risk factors. The involvement of pharmacists can serve as a reminder, but more measures or methods are still needed to raise the awareness of endocrinologists on comprehensive management and risk factor compliance.

Cardiovascular disease remains the leading cause of death in patients with diabetes. Cardiovascular disease in diabetes is multifactorial, and control of the cardiovascular risk factors leads to substantial reductions in cardiovascular events. This study suggests that clinical practice has insufficient interventions for cardiovascular-related risk factors, and there is a large gap in the guidelines, which may increase the risk of adverse cardiovascular outcomes and lead to elevated health risks for patients and increased burden of medical public health expenditures, among others. There are some limitations to the study, it is only a single-center study, and the sample size included is limited, so in-depth multi-center studies with larger sample sizes should be done in the future.

In this study, we analyzed the management of blood glucose, blood pressure, lipids and the application of antiplatelet drugs in inpatients with T2DM, which can reveal the deficiencies of pharmacological management and help us to develop a more targeted management model that is more suitable for patients with T2DM in China. Only by controlling multiple risk factors up to standard can we reduce the risk of adverse cardiovascular and cerebrovascular events. The risk of adverse cardiovascular and cerebrovascular outcomes in patients with diabetes can only be reduced by controlling multiple risk factors.

## Data Availability Statement

The original contributions presented in the study are included in the article/supplementary material. Further inquiries can be directed to the corresponding authors.

## Author Contributions

X-FW, JL, S-QL, S-SX researched and collected data. XC, L-MD, Z-HS, X-LW, JB analysed the date. Z-HS, X-LW, S-SX wrote the manuscript. J-BZ and CZ reviewed and revised the manuscript. X-LW and Z-HS are the guarantor of this work and, as such, had full access to all the data in the study and take responsibility for the integrity of the data and the accuracy of the data analysis. All authors contributed to the article and approved the submitted version.

## Funding

This work was supported by the National Natural Science Foundation of China (grant numbers 82070851, 81870556), the Beijing Municipal Administration of Hospitals’ Youth Program (grant number QML20170204), and Excellent Talents in the Dongcheng District of Beijing. The authors are solely responsible for the design and conduct of this study, all study analyses, the drafting and editing of the manuscript, and its final contents. b. Financial Disclosures: No financial disclosures.

## Conflict of Interest

The authors declare that the research was conducted in the absence of any commercial or financial relationships that could be construed as a potential conflict of interest.

## Publisher’s Note

All claims expressed in this article are solely those of the authors and do not necessarily represent those of their affiliated organizations, or those of the publisher, the editors and the reviewers. Any product that may be evaluated in this article, or claim that may be made by its manufacturer, is not guaranteed or endorsed by the publisher.
